# Hydroxyl-Terminated Saponified Natural Rubber Based on the H_2_O_2_/P25-TiO_2_ Powder/UVC-Irradiation System

**DOI:** 10.3390/polym13081319

**Published:** 2021-04-17

**Authors:** Supinya Nijpanich, Adun Nimpaiboon, Porntip Rojruthai, Jitladda Sakdapipanich

**Affiliations:** 1 Department of Chemistry and Center of Excellence for Innovation in Chemistry, Faculty of Science, Mahidol University, Nakhon Pathom 73170, Thailand; supinya@slri.or.th; 2 Synchrotron Light Research Institute, Nakhon Ratchasima 30000, Thailand; 3 Rubber Technology Research Centre (RTEC), Faculty of Science, Mahidol University, Nakhon Pathom 73170, Thailand; adun.nim@mahidol.ac.th; 4 Division of Chemical Industrial Process and Environment, Faculty of Science, Energy and Environment, King Mongkut’s University of Technology North Bangkok, Rayong 21120, Thailand; porntip.r@sciee.kmutnb.ac.th

**Keywords:** natural rubber, functionalized low-molecular-weight natural rubber, photochemical degradation process, titanium oxide (TiO_2_)

## Abstract

Natural rubber (NR), a long-chain hydrocarbon polymer mostly consisting of *cis*-1,4-polyisoprene units, has a high molecular weight (MW) and viscosity, enabling it to show excellent physical properties. However, NR has no reactive functional group, making it difficult to react with other molecules, especially in manufacturing processes. The functionalized low-molecular-weight NR (FLNR) is a requirement to disperse ingredients into the rubber adequately. Here, the FLNR was prepared by a photochemical degradation process under UVC-irradiation in the presence of H_2_O_2_ using P25-titanium oxide (TiO_2_) powder as a photocatalyst. The optimum condition for the preparation of FLNR was the use of 2.0 g of TiO_2_ powder per 100 g of rubber and H_2_O_2_ at 20% *w*/*w* under UVC-irradiation for 5 h. The hydroxyl groups were found on the NR chains due to the chain-scission of polyisoprene chains and hydroxyl radicals in the system. The weight average MW of NR decreased from 12.6 × 10^5^ to 0.6 × 10^5^ gmol^−1^, while the number average MW decreased from 3.3 × 10^5^ to 0.1 × 10^5^ gmol^−1^.

## 1. Introduction

Natural rubber (NR) derived from the *Hevea brasiliensis* tree is a long chain hydrocarbon polymer composed of 94% *cis*-1,4-polyisoprene and 6% non-rubber components, including lipids, proteins, and inorganic constituents [1]. It has been extensively used in various applications, such as tire, medical glove, and condoms, since it provides excellent physical properties, resilience, strength, and fatigue resistance [2]. On the other hand, the very high molecular weight (MW) and hydrocarbon nature make it difficult to process and compatibilize NR with fillers or other molecules and significantly limits its chain-end usage. Several efforts to modify some NR properties by reducing its MW and introducing a certain reactive functional group at chain-ends have been reported. A shorter and functionalized NR product, called functionalized low-molecular-weight NR (FLNR), has been applied in plasticizers [3], compatibilizers [4,5], and adhesive materials [6], as well as precursors for chain extension [7,8,9] and grafting [10]. For instance, Isa et al. prepared the epoxidized NR in the latex state using sodium nitrite (NaNO_2_) as a reducing agent [11]. They found that the chain-scission of the NR chain occurred parallel with the epoxidation. Both hydroxyl and epoxide groups were observed in the degraded NR, and the degradation was influenced by temperature and reaction time. Ibrahim et al. functionalized the liquid NR via an oxidative degradation process for 24 h or 48 h [12,13]. Nevertheless, these methods require a toxic chemical and a long reaction time.

In 1988, Ravindran et al. reported the preparation of hydroxyl-terminated liquid NR via the photochemical degradation in the presence of H_2_O_2_ under ultraviolet (UV) irradiation from a medium-pressure mercury vapor lamp and sunlight [14]. Comparing these methods, the photochemical degradation method seems more promising than other techniques due to its clean and environmentally friendly method, low in energy, and non-toxic reagent consumption.

**Figure 1:**
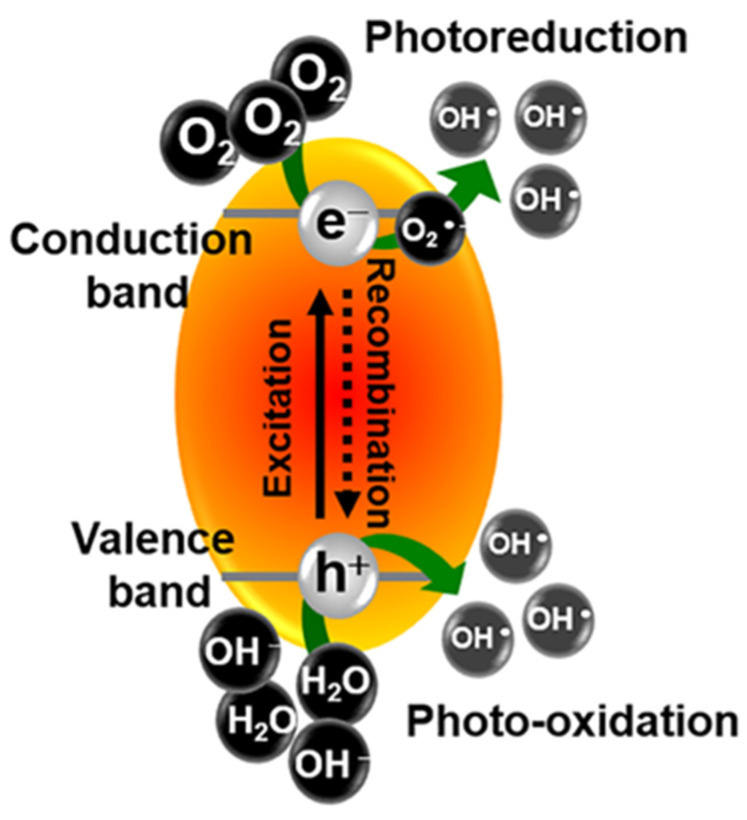
Schematic illustration of the photo-generation of charge carriers in a photocatalyst.

Previously, we reported the successful preparation of functionalized styrene-butadiene rubber and skim latex by a photocatalytic degradation process using a nanometric photocatalyst in a TiO_2_-film-coated Petri dish under ultraviolet irradiation [15]. The photocatalytic reaction on the TiO_2_ surface begins when it adsorbs light with an energy higher than its bandgap energy. The electron (e^−^) in the valence band will be excited and jump to the conduction band, leaving a positively charged hole (h^+^). When the e^−^ and h^+^ are transferred to reactive species, i.e., O_2_ or H_2_O, adsorbed on the TiO_2_ surface, the hydroxyl radicals (OH∙) can be generated, as illustrated in Figure 1.

The generated reactive oxygen species play an important role in the decomposition of NR. It is well known that the high surface area of a photocatalyst influences its photocatalytic ability [16]. Although the FLNR could be obtained in our previous work, the reaction site was limited to the size of a Petri dish and the procedure to prepare the TiO_2_-coated Petri dish was complicated. In addition, based on the NR particle structure reported by Nawamawat et al. [17], the NR particle surface was surrounded by a layer made up of proteins and phospholipids, stabilizing the hydrophobic polyisoprene chains. However, the mixed layer of phospholipids and proteins stabilized the rubber chains and obstructed the NR chains from other molecules once the photocatalytic degradation process occurred [13]. This resulted in a low reaction performance on the NR chains.

In this work, AEROXIDE^®^ P25-TiO_2_ powder was utilized as a photocatalyst instead of a TiO_2_-coated Petri dish to enhance the reaction site due to its large specific surface area and to minimize the photocatalyst preparing step. Furthermore, a mixed layer of proteins and phospholipids surrounding the surface of NR was also preliminarily eliminated from NR particles by saponification, giving a so-called saponified NR (SPNR) latex. Then, it was used as a starting material to prepare FLNR latex in the presence of H_2_O_2_ under UVC-irradiation using AEROXIDE^®^ P25-TiO_2_ powder as a photocatalyst. The optimum condition was investigated, including the effect of TiO_2_ powder as well as UVC-irradiation time. The prepared FLNR was further characterized using Fourier-Transform Infrared spectroscopy (FT-IR), Gel Permeation Chromatography (GPC), and Nuclear Magnetic Resonance spectroscopy (NMR).

## 2. Materials and Methods

### 2.1. Materials

The NR latex used in this study was high ammonia (HA) latex supplied from Thai rubber latex Group Public Co., Ltd. (Bang Phli District, Thailand). Hydrogen peroxide (H_2_O_2_, 30% *w*/*w*) was purchased from Merck. Methanol (CH_3_OH), sodium hydroxide (NaOH), tetrahydrofuran (THF), and toluene were purchased from Labscan^®^. P25-TiO_2_ (AEROXIDE^®^) powder was kindly supported by Evonik industries. All chemicals are analytical (AR) grades and were used as received, without further purification.

### 2.2. Methods

#### 2.2.1. Preparation of SPNR

Prior to the saponification process, HA latex was diluted with distilled water from 60% dry rubber content (DRC) to 30% DRC in the presence of 1% *w*/*v* SDS. Then, latex was saponified with a 3.0% *w*/*v* NaOH solution [18]. The mixture was stirred at 70 °C for 3 h and cooled down at room temperature. The cream fraction was collected by double centrifugation in distilled water at 13,000 rpm (relative centrifugal force (rcf) = 15,115× *g*) for 30 min to remove the residual lipids and proteins. Finally, the resulting latex was diluted with distilled water again to get 10% DRC of SPNR latex.

#### 2.2.2. Preparation of FLNR

The mixtures of 30 g SPNR latex at 10% DRC, TiO_2_ powder (0, 0.25, 0.5, 1.0, 2.0 g per 100 g of rubber or phr), and H_2_O_2_ (0, 5, 10, 15, 20% *w*/*w*) were irradiated in a self-constructed UV chamber under a 80W UV lamp (λ_max_ at 253 nm) for 5 h. Then, the mixtures were centrifuged at 13,000 rpm (15,115× g) for 20 min to remove the TiO_2_ particles from the solution. The cream rubber fraction was collected into a Petri dish and dried in a vacuum oven at 40 °C for 12 h. The samples were purified by dissolving in toluene and further coagulated with CH_3_OH. The obtained rubber was dried in a vacuum oven at 40 °C for 12 h and then subjected to the characterization.

### 2.3. Characterizations

#### 2.3.1. Determination of the Crystallinity of TiO_2_ Powder

The AEROXIDE^®^ P25-TiO_2_ powder was characterized by the XRD technique using a Bruker^®^ D8 Advance X-ray diffractometer with CuK_α_ radiation (λ = 1.5406 Å) operated at the accelerating voltage and current of 40 kV and 40 mA, respectively. The measured two theta (2θ) range were 20–60, with a scanning speed of 2.0 °min^−1^.

#### 2.3.2. Determination of the Chemical Structure of the Rubber Samples

Fourier-Transform Infrared (FTIR) Spectroscopy was carried out using a JASCO FTIR-4100 spectrometer. In a typical procedure, the dried rubber was dissolved in chloroform (1% *w*/*v*) and stirred for 24 h. The obtained solution was cast and dried on a germanium (Ge) prism to get a thin film. The cast film attached to the Ge prism was then inserted into the spectrometer and measured at a resolution of 4 cm^−1^ with 100 scans to analyze the functional group of samples.

^1^H-nuclear magnetic resonance (^1^H-NMR) spectroscopy was recorded on BRUKER 500 UltraShield^TM^. 0.3–0.4% *w*/*v* of dried rubber was dissolved in *d*-chloroform (*d*-CDCl_3_). Then, the sample was subjected to a ^1^H-NMR instrument.

Gel permeation chromatography (GPC) was used to determine the MW and molecular-weight distribution (MWD). The dried rubber was firstly dissolved in THF to make a solution with a concentration of 0.05% *w*/*v*. The solution was filtered through a 0.45 µm nylon-membrane syringe filter to remove impurities contaminating the solution. After that, 100 µL of the solution was injected into the JASCO-Borwin^®^ GPC set-up equipped with RI (Jasco RI 1530) detectors using THF as an eluent at 35 °C, with a flow rate of 0.5 mL min^−1^. A column set was employed consisting of three sets of 300 × 8 mm columns in which the exclusion limits of a column packed with polystyrene-divinylbenzene gels of narrow particle size distribution were 4 × 10^5^, 5 × 10^6^, and 2 × 10^8^. MWD was calculated based on polyisoprene standards.

## 3. Results and Discussion

### 3.1. Characterization of TiO_2_ Powder

**Figure 2:**
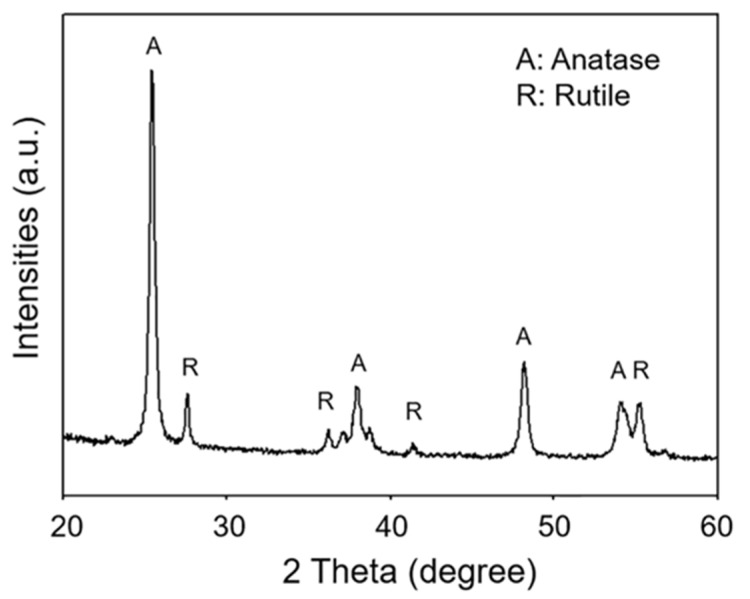
XRD pattern of P25-TiO_2_ powder.

The crystallinity of TiO_2_ powder was preliminarily investigated. The clear diffraction peak in the X-ray diffraction (XRD) pattern was observed at 2θ of 25.4°, corresponding to the (101) plane of anatase, as shown in Figure 2. Other anatase peaks were observed at 2θ of the 37.9° (004), 48.1° (200), 54.1° (105), and 55.4° (211) planes. Moreover, the rutile peaks were also observed at 2θ of the 27.5° (110), 36.1° (101), 41.4° (111), and 56.8° (220) planes. This result indicated that P25-TiO_2_ powder consisted of anatase and rutile phases. Hurum et al. proposed that the mixed-phase of anatase and rutile enhanced the photocatalytic activity of TiO_2_ powder compared with the pure phase, since the rutile e^−^ was scavenged and the e^−^-h^+^ recombination was also hindered [19].

To examine the optimum condition for the FLNR latex preparation, the effect of TiO_2_ powder content, H_2_O_2_ concentration, and UVC-irradiation time were studied. The functional groups present on the rubber chains and the change in ${\bar{M}}_{n}$ and ${\bar{M}}_{w}$ before and after functionalization, including the chemical structure of the FLNR, were also investigated.

### 3.2. Effect of the TiO_2_ Powder Content on the MW and the Structure of SPNR and FLNR 

**Figure 3:**
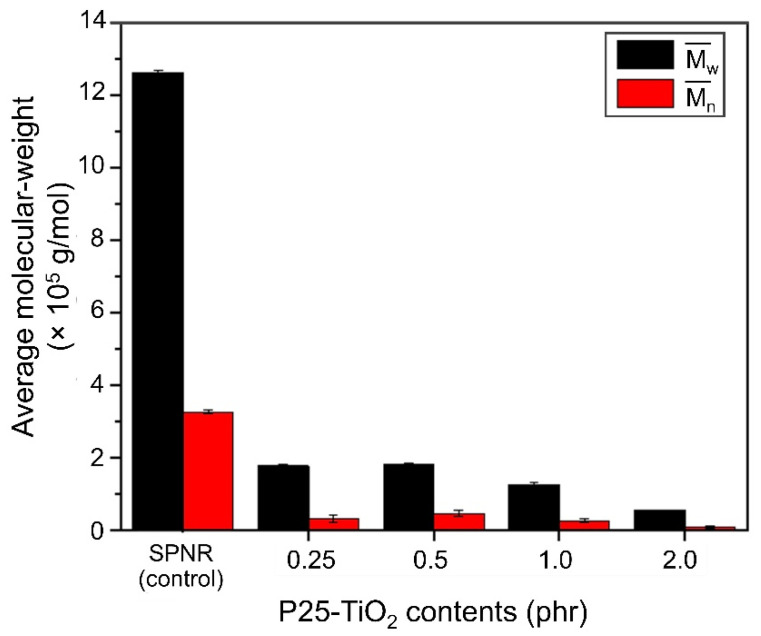
The average MW of SPNR and samples prepared by introducing various TiO_2_ powder contents in the presence of 20% *w*/*w* H_2_O_2_ after UVC-irradiation for 5 h.

**Figure 4:**
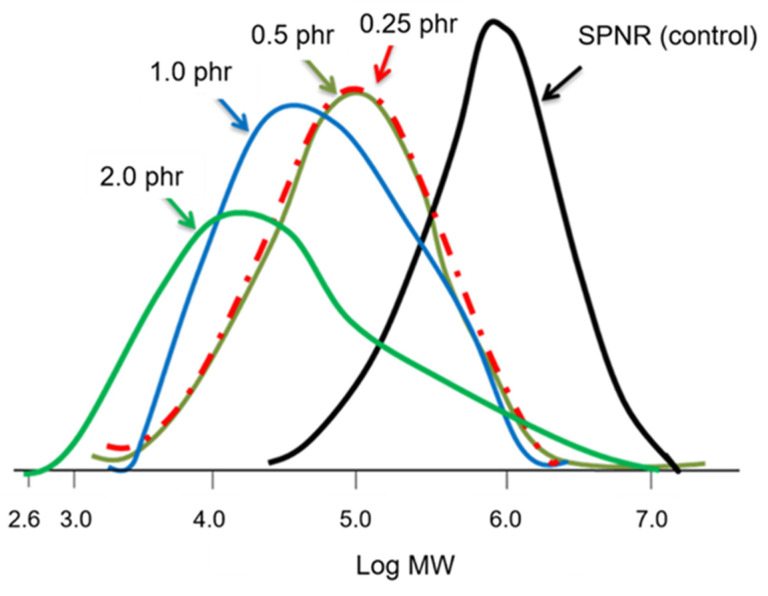
MWD of SPNR latex samples using H_2_O_2_ 20% *w*/*w* with various TiO_2_ powder contents under UVC-irradiation for 5 h.

In order that the UVC light can pass through the latex mixture efficiently, leading to an adequate energy for activating the TiO_2_ powder, the low content of TiO_2_ powder was varied between 0 and 2.0 phr. Figure 3 shows the ${\bar{M}}_{n}$ and ${\bar{M}}_{w}$ of SPNR and the samples obtained from the functionalization using different TiO_2_ contents in the presence of 20% *w*/*w* H_2_O_2_ under 5 h of UVC-irradiation. It was found that ${\bar{M}}_{n}$ and ${\bar{M}}_{w}$ dramatically decreased when the TiO_2_ powder was incorporated, and it gradually decreased with an increase of TiO_2_ content. At 2.0 phr of TiO_2_ powder, ${\bar{M}}_{n}$ and ${\bar{M}}_{w}$ dropped to their lowest levels, which were about 0.6 × 10^5^ and 0.1 × 10^5^ gmol^−1^, respectively. However, the higher possible reactive site on the NR particles’ surface is difficult to control for different NR chain lengths. It is reasonable to presume that shorter chains have a higher possibility of being broken down into a large number of low-MW NRs, resulting in the broadening of MWD after the degradation process. The MWD of the SPNR latex samples is shown in Figure 4, indicating the unimodal distribution for all samples.

**Figure 5:**
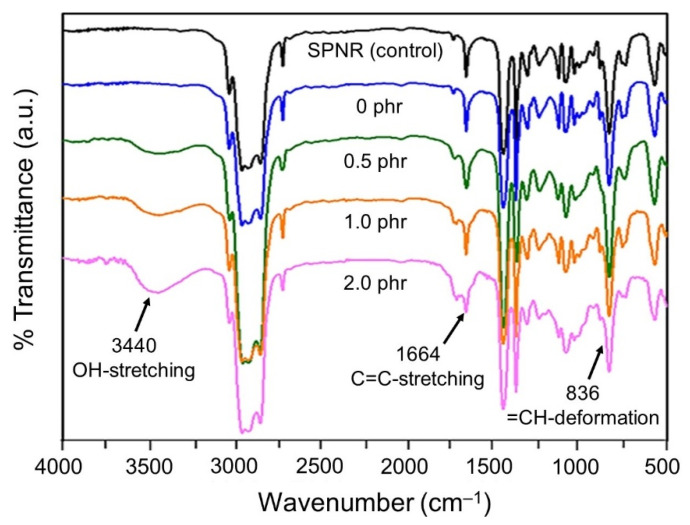
FTIR spectra of SPNR and samples with various TiO_2_ contents in the presence of 20% *w*/*w* H_2_O_2_ after UVC-irradiation for 5 h.

The functional groups on rubber chains were observed by FTIR spectroscopy. FTIR spectra of SPNR and the samples are shown in Figure 5. The important characteristic bands for NR appear around 1664 and 836 cm^−1^, assigned to the C=C stretching and =C–H deformation, respectively [20]. The new transmittance peak around 3440 cm^−1^, corresponding to the –OH group, was observed in the sample prepared in the system containing TiO_2_ powder, and the peak intensity increased with the increase of TiO_2_ content from 0 to 2.0 phr. This suggests that TiO_2_ accelerates the photochemical degradation of SPNR latex through the photocatalysis on the TiO_2_ surface. The higher the amount of TiO_2_ powder, the higher the –OH groups from the oxidation phenomena. The generated OH∙ attached to the broken points of polyisoprene chains, which shortened during the reaction, confirmed the decrease in the average MW given in Figure 3.

### 3.3. Effect of H_2_O_2_ Content on the MW and the Structure of SPNR and FLNR

**Figure 6:**
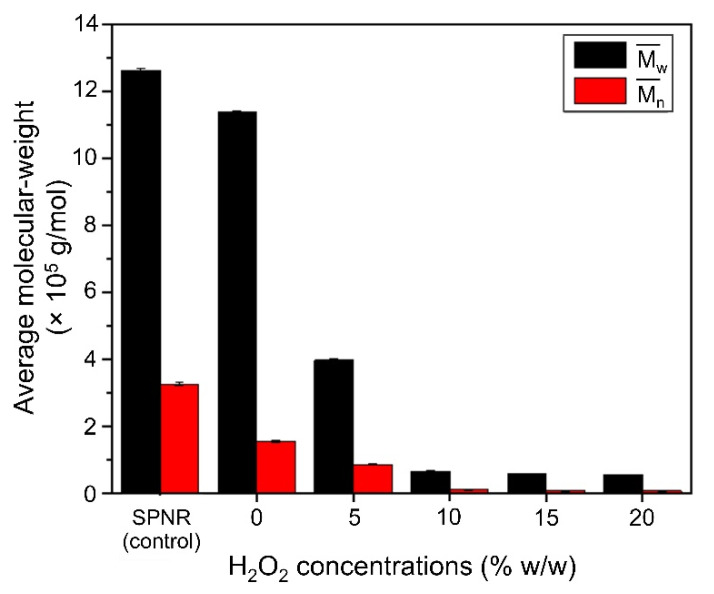
The average MW of SPNR and the samples prepared by introducing 2.0 phr of TiO_2_ powder with various H_2_O_2_ concentrations after UVC-irradiation for 5 h.

The ${\bar{M}}_{w}$ and ${\bar{M}}_{n}$ of SPNR and the samples prepared by introducing 2.0 phr of TiO_2_ powder with different H_2_O_2_ concentrations under UVC-irradiation are shown in Figure 6. It is clear that ${\bar{M}}_{n}$ and ${\bar{M}}_{w}$ slightly decreased in the presence of TiO_2_ without H_2_O_2,_ but drastically decreased when the H_2_O_2_ was introduced. Moreover, both the ${\bar{M}}_{n}$ and ${\bar{M}}_{w}$ of the samples decreased with an increase of the H_2_O_2_ concentration.

**Figure 7:**
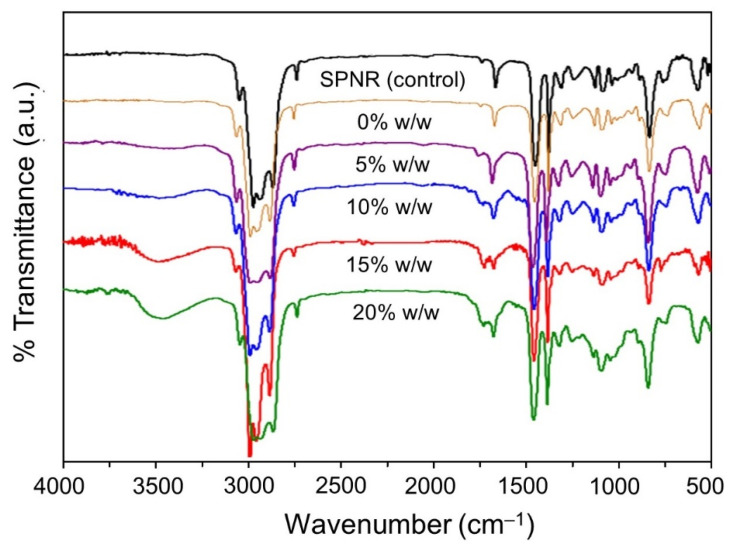
FTIR spectra of SPNR and the samples prepared by introducing 2.0 phr of TiO_2_ powder with various H_2_O_2_ concentrations after UVC-irradiation for 5 h.

Given this result, we assumed that the average MW of the samples decreased because many OH∙ took place in the system, which was generated from both TiO_2_ photocatalysis and the degradation of H_2_O_2_ under UVC-irradiation [21]. The functional groups on SPNR and the samples prepared by introducing 2.0 phr of TiO_2_ powder at different H_2_O_2_ concentrations are shown in Figure 7. The –OH group at around 3440 cm^−1^ was obviously observed when H_2_O_2_ was introduced in the system, and it increased with the increasing of H_2_O_2_ concentration. The highest –OH intensity was observed in the sample containing 20% *w*/*w* H_2_O_2_. This suggests that the increase of the –OH group resulted from the dissociation of H_2_O_2_ under UVC, apart from the oxidation of rubber via the photocatalytic reaction on the TiO_2_ surface. These observations were correlated with the result shown in Figure 6.

### 3.4. Effect of UVC-Irradiation Time on the SPNR and FLNR Structures

**Figure 8:**
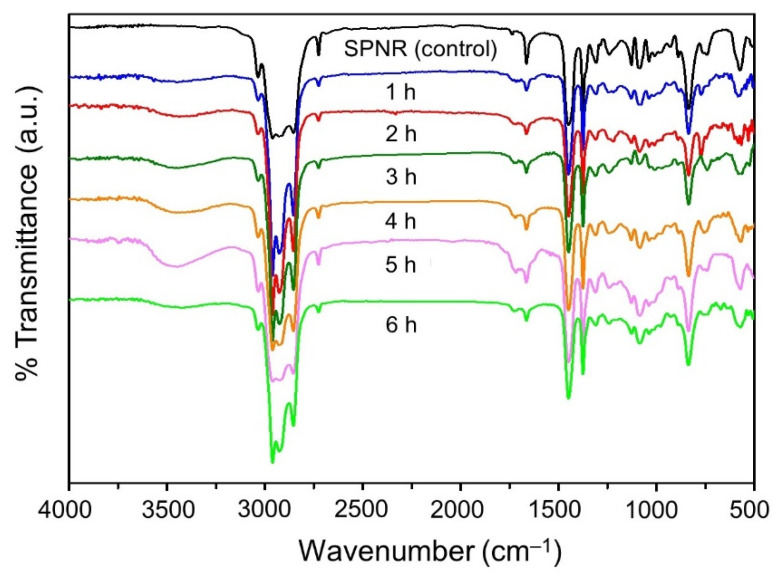
FTIR spectra of SPNR and the samples prepared by introducing 2.0 phr of TiO_2_ powder in the presence of 20% *w*/*w* H_2_O_2_ after various UVC-irradiation times.

The FTIR spectra of SPNR and the samples prepared from different UVC-irradiation time containing 2.0 phr TiO_2_ powder and 20% *w*/*w* H_2_O_2_ are exhibited in Figure 8. The transmittance peak at around 3440 cm^−1^, corresponding to -OH), increases with the increasing time of irradiation and presents the highest intensity under irradiation for 5 h. However, the -OH peak was found to decrease after UVC-irradiation for 6 h. This might be due to the occurrence of some unfavorable processes: (i) excess amounts of OH and other reactive radicals generated from the direct decomposition of H_2_O_2_ by UVC light and the formation of radicals on the TiO_2_ surface, leading to a low quantum efficiency for this method, and (ii) the recombination of h^+^ and e^−^ during the photocatalytic degradation process.

**Figure 9:**
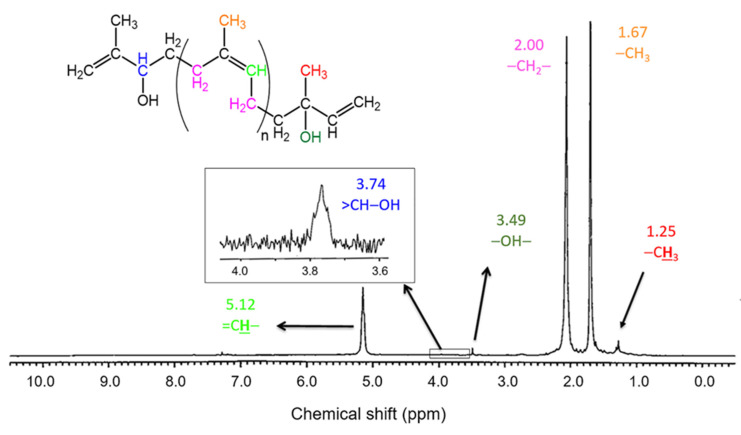
^1^H-NMR spectrum of FLNR prepared by SPNR in the presence of 2.0 phr of TiO_2_ powder and 20% *w*/*w* H_2_O_2_ under UVC-irradiation for 5 h.

The microstructure of FLNR was confirmed by the ^1^H-NMR spectrum, as shown in Figure 9. The proton peaks of NR at δ = 1.25, 2.00, and 5.12 ppm were assigned to -CH_3_, -CH_2_, and -C=CH, respectively [22]. In addition, the additional small peaks at 3.49 and 3.74 ppm were also detected, corresponding to -OH and >CH-OH, respectively. These observations implied that some -OH groups were present on the FLNR chain-ends [23,24]. The proposed structure is indicated in Figure 9.

## 4. Conclusions

The functionalized low-molecular-weight NR (FLNR) latex with a terminated hydroxyl group was successfully prepared by the photochemical degradation process of saponified NR (SPNR) latex in the presence of H_2_O_2_ using P25-TiO_2_ powder as a photocatalyst under UVC-irradiation. The elimination of the mixed layer of lipids and proteins surrounding the NR particles’ surface by the saponification was profitable on the degradation mechanism on NR chains due to the enhancement of reagents attachment. The contents of TiO_2_ powder and H_2_O_2_ as well as UVC-irradiation time were found to affect the degradation of NR. After 80W UVC-irradiation for 5 h in the presence of 20% *w*/*w* H_2_O_2_ and 2.0 phr of P25-TiO_2_ powder, the weight average MW of SPNR dramatically decreased from 12.6 × 10^5^ to 0.6 × 10^5^ gmol^−1^, while the average MW decreased from 3.3 × 10^5^ to 0.1 × 10^5^ gmol^−1^. In addition, the –OH groups were also clearly observed on the NR chains, and their contents were found to be increased with increasing TiO_2_ and H_2_O_2_ contents and UV irradiation time. The presence of –OH groups is thought to be caused by the attachment of OH∙ on the broken points of polyisoprene chains—which were shortened during the reaction, due to the H_2_O_2_ dissociation—and the photocatalysis on the TiO_2_ surface. It can be concluded that the H_2_O_2_/P25-TiO_2_ powder/UVC-irradiation system is an efficient, clean, and economical method for the preparation of FLNR latex.

## Data Availability

The data presented in this study are available on request from the corresponding author.
